# Contribution of Common Genetic Variants to Familial Aggregation of Disease and Implications for Sequencing Studies

**DOI:** 10.1371/journal.pgen.1008490

**Published:** 2019-11-15

**Authors:** Andrew Schlafly, Ruth M. Pfeiffer, Eduardo Nagore, Susana Puig, Donato Calista, Paola Ghiorzo, Chiara Menin, Maria Concetta Fargnoli, Ketty Peris, Lei Song, Tongwu Zhang, Jianxin Shi, Maria Teresa Landi, Joshua Neil Sampson

**Affiliations:** 1 Integrative Tumor Epidemiology Branch: Division of Cancer Epidemiology and GeneticsNational Cancer Institute, Rockville, Maryland, United States of America; 2 Perelman School of Medicine, University of Pennsylvania, Philadelphia, Pennsylvania, United States of America; 3 Biostatistics Branch: Division of Cancer Epidemiology and Genetics, National Cancer Institute, Rockville, Maryland, United States of America; 4 Department of Dermatology, Instituto Valenciano de Oncología, València, Spain; 5 Dermatology Department, Melanoma Unit, Hospital Clínic de Barcelona, IDIBAPS, Universitat de Barcelona, Centro de Investigación Biomédica en Red de Enfermedades Raras (CIBERER), Barcelona, Spain; 6 Department of Dermatology, Maurizio Bufalini Hospital, Cesena, Italy; 7 Genetics of Rare Cancers, Department of Internal Medicine (DiMI), University of Genoa and Ospedale Policlinico San Martino Genoa, Genoa, Italy; 8 Immunology and Molecular Oncology Unit, Veneto Institute of Oncology IOV—IRCCS, Padua, Italy; 9 Department of Dermatology, Department of Biotechnological and Applied Clinical Sciences, University of L’Aquila, L’Aquila, Italy; 10 Institute of Dermatology, Catholic University, Rome, Italy; 11 Fondazione Policlinico Universitario A. Gemelli, IRCCS, Rome, Italy; Newcastle University, UNITED KINGDOM

## Abstract

Despite genetics being accepted as the primary cause of familial aggregation for most diseases, it is still unclear whether afflicted families are likely to share a single highly penetrant rare variant, many minimally penetrant common variants, or a combination of the two types of variants. We therefore use recent estimates of SNP heritability and the liability threshold model to estimate the proportion of afflicted families likely to carry a rare, causal variant. We then show that Polygenic Risk Scores (PRS) may be useful for identifying families likely to carry such a rare variant and therefore for prioritizing families to include in sequencing studies with that aim. Specifically, we introduce a new statistic that estimates the proportion of individuals carrying causal rare variants based on the family structure, disease pattern, and PRS of genotyped individuals. Finally, we consider data from the MelaNostrum consortium and show that, despite an estimated PRS heritability of only 0.05 for melanoma, families carrying putative causal variants had a statistically significantly lower PRS, supporting the idea that PRS prioritization may be a useful future tool. However, it will be important to evaluate whether the presence of rare mendelian variants are generally associated with the proposed test statistic or lower PRS in future and larger studies.

## Author summary

Multiple members in a family can be diagnosed with the same disease. In such families, genetics may be a significant factor in disease risk. However, it remains unclear whether such familial aggregation of disease is likely due to a single highly penetrant rare variant (HPRV), many minimally penetrant common variants, or a combination of the two types of variants. We therefore use recent estimates of SNP heritability and the liability threshold model to estimate the proportion of afflicted families likely to carry a rare, causal variant. We then show that Polygenic Risk Scores (PRS) may be useful for identifying families likely to carry such a rare variant and introduce a related statistic that can be used to select families for sequencing studies trying to identify HPRV.

## Introduction

Genetics is the primary cause for familial aggregation of disease [1]. In afflicted families, members often share either a single highly penetrant rare genetic variant (HPRV) [2], a large number of minimally penetrant common variants [3], or both[4]. In this article we have two objectives. First, we show that a non-negligible fraction of familial aggregation may be attributable to common variants. Second, given this fact, we show that sequencing studies should try to select families with members having low Polygenic Risk Scores (PRS) (i.e. few risk alleles at common variants) when the study’s objective is to identify new highly penetrant rare variants. Importantly, the genotyping needed for calculating PRS is usually inexpensive relative to full sequencing.

The importance of common variation in the familial aggregation of disease is demonstrated by the large estimates of SNP heritability [5] and the elevated PRS in members of afflicted families. High PRS has already been reported for families with migraines [6], dyslipidemia [7, 8], Crohn’s disease [9], Alzheimer’s [10], schizophrenia [11], and breast cancer with [12] and without [13–15] BRCA mutations. The increased number of common risk variants can either be solely responsible for the familial aggregation of disease or can exaggerate [16] the penetrance of an HPRV. In the first half of this paper, we suggest that for many diseases with high SNP heritability and a prevalence above 1%, a non-negligible proportion of afflicted families may not harbor an HPRV.

As only a fraction of afflicted families will have an HPRV, sequencing studies intended to identify new HPRV should preferentially select those families most likely to carry such a variant. For our discussion, we consider a sequencing study that compares the proportion of individuals in the selected families with a specific HPRV with the proportion of individuals in a large, biobank-sized, set of controls carrying that HPRV. Therefore, the power for this illustrative study, as well for many other types of sequencing studies, will be determined by the proportion of tested family members with the HPRV. As initially discussed in Jostins [17], such preferential selection can be achieved by selecting families with the lowest PRS. Therefore, in the second half of this paper, we define a PRS-based statistic for prioritizing families and explore the potential for preferential selection to increase the statistical power of the illustrative study. We acknowledge that prioritization may only be useful in the near future, while the genotyping needed to obtain the PRS is actionably less expensive than sequencing or we continue to work with families that have already been genotyped. Furthermore, we use results from a recent Whole-Exome-Sequencing (WES) Study of Melanoma Families from the MelaNostrum consortium to provide an example where families with putative HPRV appear to have lower PRS than other afflicted families.

Our article proceeds as follows. In the results section, we describe HPRV prevalence in afflicted families, demonstrate the potential for PRS-guided selection to increase study power, and discuss the relationship between PRS and the presence of HPRV in the MelaNostrum consortium. In the discussion section, we review the key points, relate our approach to the literature, and suggest next steps. In the methods section, we summarize notation, define our PRS statistic, provide full details on the scenarios used for evaluating familial aggregation, and describe the MelaNostrum consortium. Finally, it is important to note that this article, with its theory grounded in a simple liability threshold model, is intended to provide only rough approximations of HPRV prevalence and study power. However, despite the dependence on this model, we believe that our conclusions, at least qualitatively if not quantitively, are valid.

## Results

**Fig 1:**
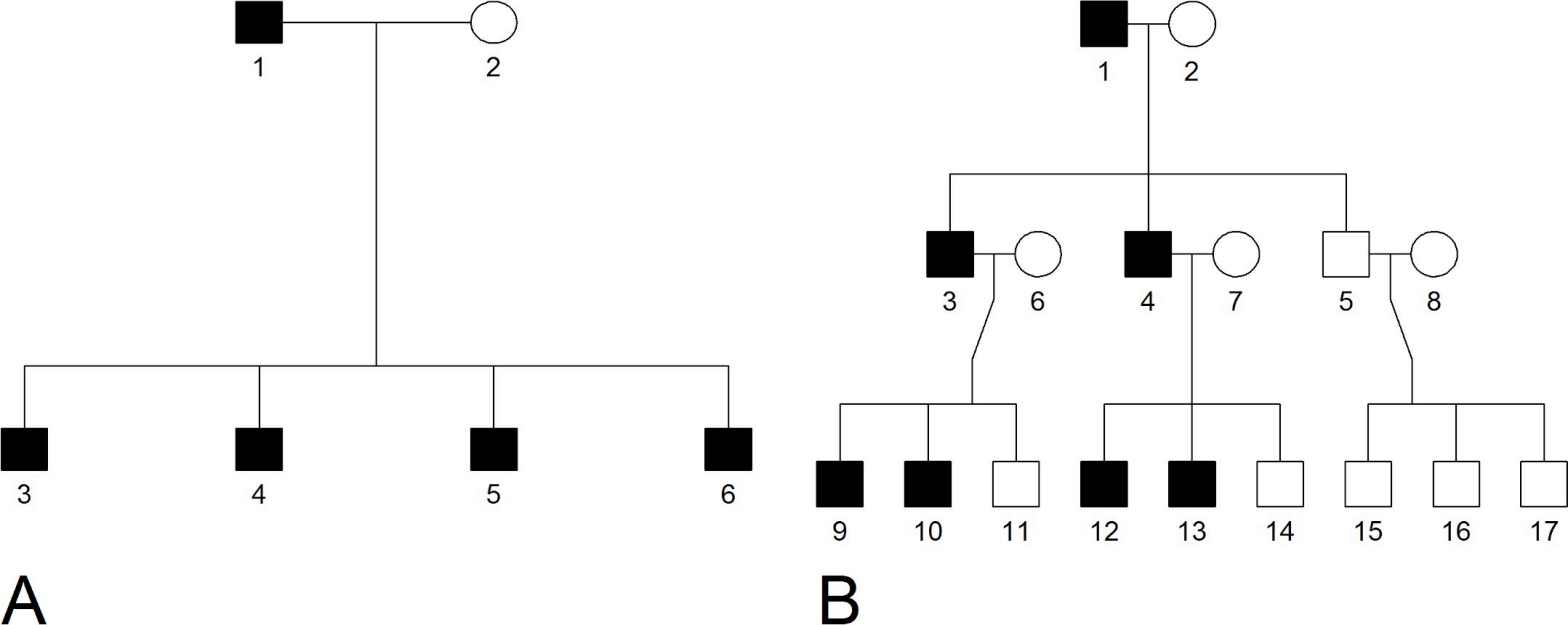
Examples family structure. (A) This example of the two-generation family structure has a founding couple with $n_{1}^{D}=4$ affected children and $n_{1}^{T}=4$ total children. (B) This example of the three-generation family structure has a founding couple with $n_{1}^{D}=2$ affected children, $n_{1}^{T}=3$ total children, $n_{2}^{D}=4$ affected grandchildren, and $n_{2}^{T}=9$ total grandchildren.

We start by describing how the specific features of a disease affect the proportion of affected family members expected to carry an HPRV and the ability of our proposed PRS-based statistic to identify those families with members carrying an HPRV. Specifically, we consider these relationships in affected sibships (e.g. bottom level of Fig 1A) and multi-generational families (e.g. bottom two levels of Fig 1B). We explore the relationships using the simulations described in the Methods section and, for reference, we note that the notation used to describe the evaluated features are fully listed in the Methods: Notation section.

**Fig 2:**
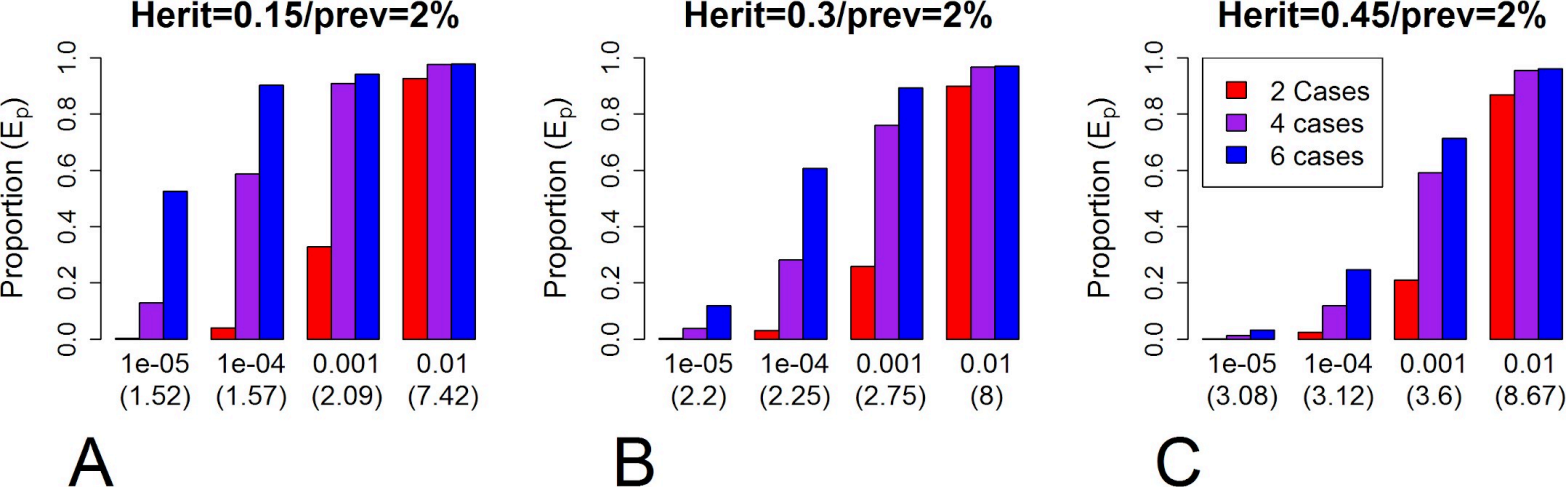
Proportion carrying HPRV in affected sibships. The proportion (*E*_*p*_) of individuals carrying a highly penetrant rare variant (HPRV) in affected sibships for a disease with a 2% prevalence (100 × π*) in the population. Panels A, B, and C represent different values of polygenic heritability (A: $\sigma_{P}^{2}=0.15$, B: $\sigma_{P}^{2}=0.30$, C: $\sigma_{P}^{2}=0.45$). Within a panel, the four sets of bars represent different MAF (*p*_*G*_ = 0.00001, 0.0001, 0.001, or 0.01; note, the number in parenthesis is the resulting Sibling Relative Risk). Within a set of bars, the colors represent the different number of children (red: $n_{1}^{T}=n_{1}^{D}=2$, purple: $n_{1}^{T}=n_{1}^{D}=4$, blue: $n_{1}^{T}=n_{1}^{D}=6$). See Supporting Information: S2 Fig for diseases with other prevalences.

**Fig 3:**
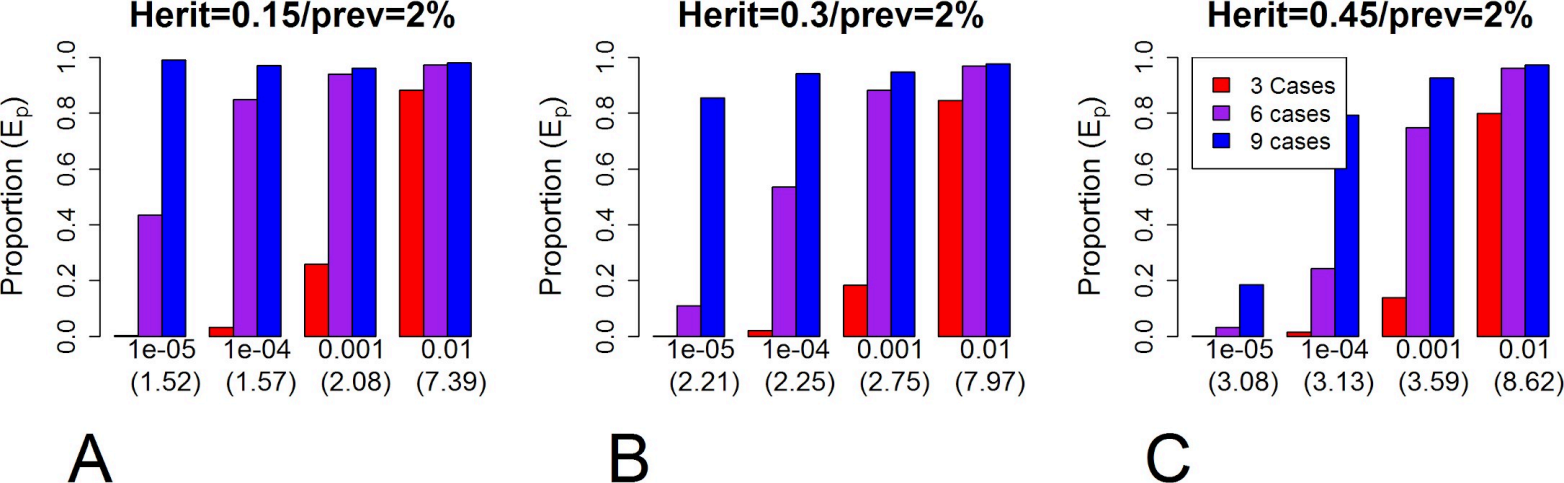
Proportion carrying HPRV in multi-generational families. The proportion (*E*_*p*_) of individuals carrying a highly penetrant rare variant (HPRV) in affected multi-generational families for a disease with a 2% prevalence (100×π*) in the population. Panels A, B, and C represent different values of polygenic heritability (A: $\sigma_{P}^{2}=0.15$, B: $\sigma_{P}^{2}=0.30$, C: $\sigma_{P}^{2}=0.45$). Within a panel, the four sets of bars represent different MAF (*p*_*G*_ = 0.00001, 0.0001, 0.001, or 0.01; note, the number in parenthesis is the resulting Sibling Relative Risk). Within a set of bars, the colors represent the different numbers of total affected individuals (red: $n_{1}^{D}+n_{2}^{D}=3$, purple: $n_{1}^{D}+n_{2}^{D}=6$, blue: $n_{1}^{D}+n_{2}^{D}=9$). See Supporting Information: S3 Fig for diseases with other prevalences.

The first result is that the proportion of affected family members carrying an HPRV depends on the genetic architecture of the disease. The proportion (*E*_*p*_) carrying an HPRV increases with increasing HPRV MAF (*p*_*G*_), penetrance in individuals carrying a single HPRV (*π*_1_), and disease frequency in the family; and the proportion decreases with increasing disease prevalence (π^*^) and polygenic heritability ($\sigma_{P}^{2}$). We note, at this time, the polygenic heritability is usually an order of magnitude higher than the PRS heritability ($\sigma_{S}^{2}$). Figs 2 and S2 show examples for affected sibships and Figs 3 and S3 show examples for multi-generational families. For example, consider diseases that have a prevalence of π* = 0.02 and a heritability of $\sigma_{P}^{2}=0.3$ (e.g. [5] ischemic stroke, Barret’s esophagus, schizophrenia). In sibships where all four siblings develop disease, approximately 5%, 30%, and 80% of the affected siblings will have an HPRV if the total MAF of HPRVs is 0.00001, 0.0001, and 0.001 (Fig 2B) in our evaluated scenarios. Note, the true MAF is unknown. In multi-generation families ($n_{1}^{T}=3$, $n_{2}^{T}=9$; with $n_{g}^{T}$ being the number of biologically related individuals in the g^th^ generation) where 6 of the 12 relevant individuals have disease, approximately 10%, 60%, and 90% of cases carry an HPRV when the MAF is 0.00001, 0.001, and 0.001 (Fig 3B). Note, percentages decrease dramatically when the prevalence increases to 5% (S2 and S3 Figs).

**Fig 4:**
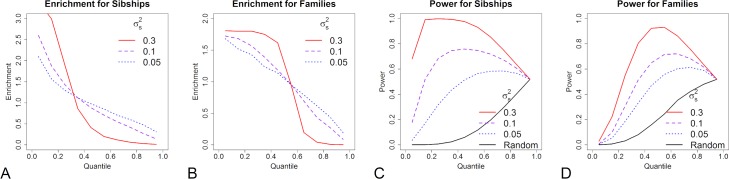
Enrichment and power to detect HPRV by the PRS statistic. Panels A and B show the enrichment (M) of HPRV as a function of the PRS statistic for a sibship with four affected individuals and a multi-generational family with six affected individuals. The X-axis is the quantile of the statistic (i.e. 0.1 represents a family at the top 10^th^ percentile). Panels C and D show the power (*β*) to detect an association with the HPRV when a sibship has four affected individuals and when a multi-generational family has six affected individuals. The X-axis is the quantile of the statistic (i.e. 0.1. represents a study where we select the 10% of affected families with the highest PRS statistic). In all four panels, the color indicates the PRS heritability (red, purple, blue indicates $\sigma_{S}^{2}=0.3$, 0.1, and 0.05 respectively), the MAF is fixed at *p*_*G*_ = 0.0001, the polygenic heritability is fixed at $\sigma_{P}^{2}=0.3$, and the disease prevalence is fixed at 100π* = 2%. See Supporting Information: S4 Fig for other diseases with other prevalences and HPRV MAFs.

The second result is that families with larger values of our newly proposed test statistic, *T*_*i*_, will be more likely to include individuals with an HPRV. Although the formal definition is postponed until the Methods, *T*_*i*_ reflects the expected number of HPRV per affected individual in a given family based on the pattern of disease and polygenic risk scores (PRS). We note that although *T*_*i*_ can be used to rank families, the ability of that ranking to differentiate families with and without an HPRV strongly depends on both the genetic architecture and predictive accuracy of the PRS. As an example, we describe HPRV enrichment in diseases that have a total polygenic heritability of $\sigma_{P}^{2}=0.3$, a prevalence of π* = 0.02, and an HPRV MAF of *p*_*G*_ = 0.0001 (Fig 4). Here, enrichment, M, is defined as a ratio, where we divide the average number of HPRV in afflicted families at a given value of T_i_ by the average number of HPRV in all afflicted families. For an affected sibship with four cases, families with *T*_*i*_ at the top 20^th^ percentile have a 2.5-fold enrichment in HPRV, as compared to the average affected sibship, when the PRS heritability is high ($\sigma_{S}^{2}=\sigma_{P}^{2}=0.3$) and a 1.5-fold enrichment when the PRS heritability is more modest ($\sigma_{S}^{2}=0.05$). Here, $\sigma_{S}^{2}$ denotes the polygenic heritability captured by the PRS. For the multi-generational family with six cases, families with *T*_*i*_ at the top 20^th^ percentile have a 1.8-fold enrichment and a 1.4-enrichment in HPRV when the PRS heritability is 0.3 and 0.05 respectively. We note that relatively high enrichment for HPRV even when the $\sigma_{S}^{2}$ is small is consistent with the behavior of the liability threshold model (see S1 Fig) which ensures individuals with low PRS are unlikely to have the disease.

The next result is that studies which preferentially select families with higher values of our proposed statistic can have increased power to detect associated HPRV. Details of the assumed study design and statistical test are presented in the Methods Section Briefly, we first assume that we select a group of families with a specific family structure for the sequencing study. Then, we compare the proportion of afflicted family members carrying a specific HPRV to the proportion of a large set of unaffected controls carrying that HPRV. Again, we consider the ideal example with $\sigma_{P}^{2}=0.3$, π* = 0.02, and *p*_*G*_ = 0.0001 (Fig 4). Note, for the discussed families, these parameters are ideal because about 50% of the families carry an HPRV and therefore intelligent selection can be beneficial. For an affected sibship with four cases or the multi-generational family with 6 cases, a study that selects families with *T*_*i*_ in the top 20^th^ percentile has notably higher power to detect a statistically significant association than a study that randomly selects the same number of families, even when the PRS heritability is modest ($\sigma_{S}^{2}=0.05$). Note, if using all families results in a power of approximately 0.5, it is expected that a study using only a randomly selected 20% of the families yields a power near 0. However, a study that enriches the HPRV in that 20% of subjects by a factor of 1.5–2.0x (i.e. selects families based on *T*_*i*_) results in approximately 10–20% power to detect a HPRV. Moreover, when that enrichment approaches 3x (e.g. $\sigma_{S}^{2}=\sigma_{P}^{2}=0.3$ in affected sibships), the power using only the enriched subset can exceed that using all subjects. We note that for other examples, especially those where nearly all affected individuals carry the HPRV, selection by the PRS statistic can, at best, only modestly improve power.

**Fig 5:**
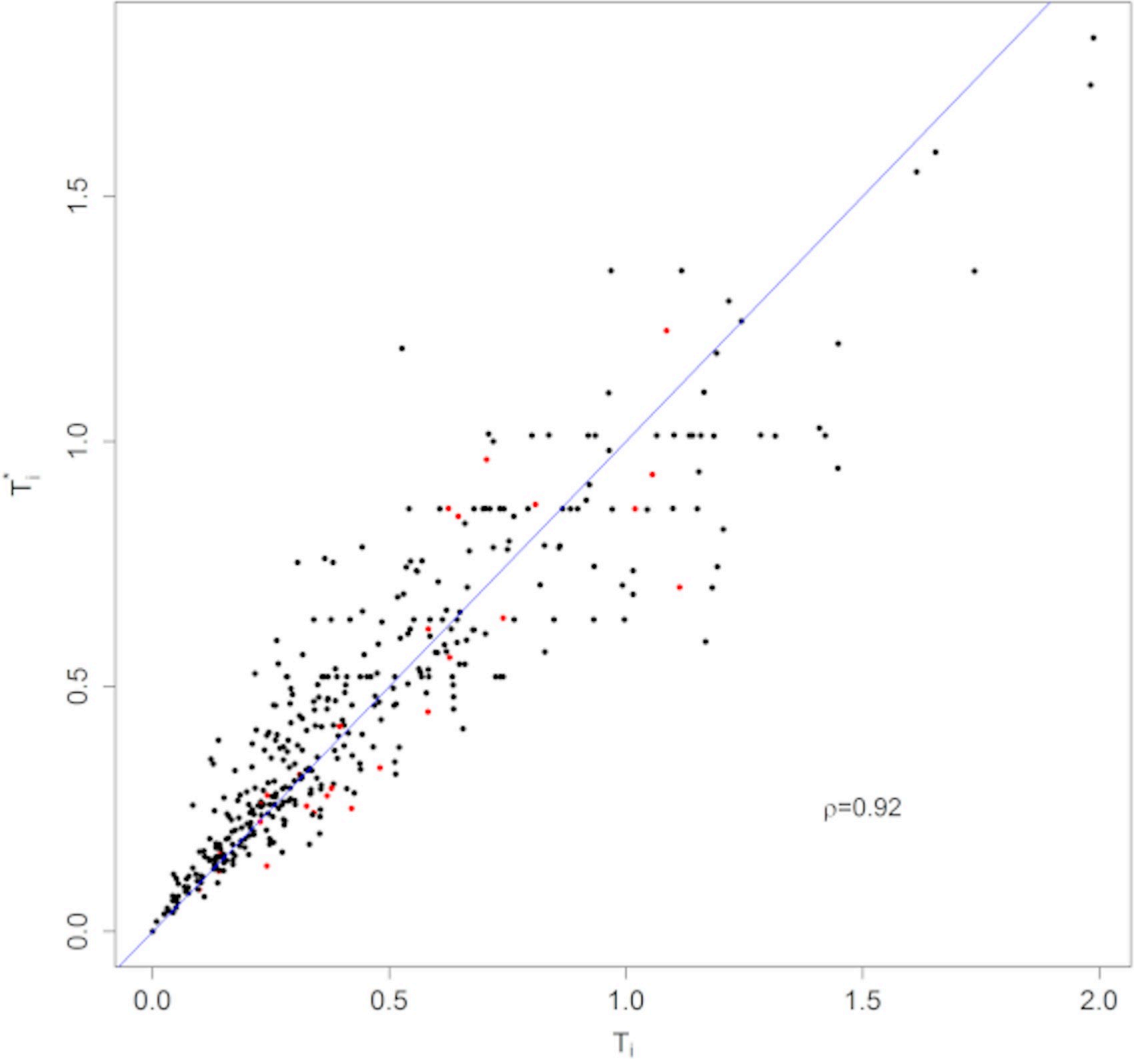
This figure compares the test statistic, *T*_*i*_, that accounts for PRS and the test statistic, $T_{i}^{*}$, that does not account for PRS in the 404 families with Melanoma. Each dot represents one family and red dots indicate families with an HPRV. The spearmen correlation (ρ) between *T*_*i*_ and $T_{i}^{*}$ is 0.92.

Finally, we evaluated the MelaNostrum families. In this study, there are 229 families from Italy and 175 families from Spain with at least one melanoma case genotyped and sequenced. The median number of individuals per family is 11 and the corresponding interquartile range (IQR) is 6–19 individuals per family. The median number of cases per family is 3 with 34% and 15% of families having at least 4 and 6 cases per family, respectively. The total number of genotyped individuals is 990, the number of genotyped cases is 711, and the number of sequenced cases is 606. We identified 29 families where at least one affected individual had a rare potentially deleterious variant, as defined in the Methods, from *ACD* (3 families), *BAP1* (2), *CDKN2A* (8), *POT1* (11), *TERF2IP* (3), and *TERT* (2). We found that the mean family PRS (i.e. the average PRS over all affected and genotyped relatives) in these 29 families was significantly lower than the mean PRS in the 368 families without a known variant, 0.09 vs 0.55 (p-value = 0.01 from t-test adjusted for country). However, we did not find the family structure or disease pattern to be related to HPRV status. Even simple metrics, such as the number or proportion of family members with disease, did not significantly differ by HPRV status. Therefore, not surprisingly, the test statistic, *T*_*i*_ was similar in the two sets of families with the mean (Standard Error, SE) in families with and without an HPRV being 0.51 (0.05) and 0.44(0.02) respectively. Finally, we note that in this example, the PRS provided only modest contributions to *T*_*i*_ given the limited number of genotyped individuals in each family (Fig 5) and that the spearman correlation coefficient between the test statistics, *T*_*i*_ and $T_{i}^{*}$, that consider and do not consider PRS was 0.92.

## Discussion

Genetics is the primary cause of the familial aggregation of diseases. Either sharing a highly penetrant rare variant (HPRV), risk alleles at multiple common variants, or a combination of both can lead to numerous cases of a disease within a family. In this paper, we aimed to describe the proportion of afflicted families likely to carry an HPRV, evaluate the ability for polygenic risk scores (PRS) to identify those families carrying an HPRV, and to offer a test statistic for prioritizing families for sequencing studies.

The first message is that for diseases with a prevalence > 1%, a proportion of afflicted families will not carry an HPRV. Unfortunately, as the number or penetrance of HPRVs for any disease is unknown, we cannot yet predict the true proportion of afflicted families who will carry HPRV. The purpose of this paper is only to show that, at least in some scenarios, there may be a significant proportion of families who do not carry an HPRV. In the future, as we continue to collect genetic information about afflicted families and new HPRVs, we might be able to postulate which of the simulated scenarios most likely reflects truth. Currently, we are limited to combining observed sibling relative risks, estimates of polygenic heritability, and disease specific knowledge of genetics to determine the realistic boundaries of possible models. We note that for rare diseases (e.g. disease prevalence 0.5%), our simulations suggest that it is difficult to obtain significant familial enrichment (e.g. affected sibships with four siblings) without the presence of an HPRV.

The second message is PRS can be used to identify those families most likely to be harboring an HPRV. Although this was expected, the surprising finding was that families with a low average PRS tended to be significantly enriched for HPRV even when the heritability explained by the PRS was relatively low ($\sigma_{S}^{2}=0.05$), suggesting that current versions of PRS could be potentially useful in selecting families for sequencing studies for non-rare diseases. This is important, since the cost per sample of SNP genotyping is much lower than that for whole exome sequencing. However, before promoting its usage, it would be important to show, in multiple studies, that the PRS are lower in families carrying HPRV. MelaNostrum would be one example, albeit with limited sample size, to suggest that families with an HPRV have lower PRS, despite melanoma being a relatively rare disease in these Mediterranean populations (age standardized incidence rate per 100,000 persons = 6.9 and 11.4 in Spain and Italy, respectively[18]). However, the newly developed statistic, which in theory should be more strongly correlated with the presence of an HPRV, did not differ across the two sets of families with and without an HPRV. We expect that our statistic will perform better in future studies, but it is worth considering why the statistic likely failed to differentiate families with and without an HPRV in MelaNostrum. First, we note that our statistic combines two types of information, the disease pattern in the family and the PRS. The first type, or disease pattern, is a time-tested means for identifying families carrying an HPRV; families with a large number of afflicted individuals tend to carry HPRV. The second type, or PRS, is the comparatively novel information used for identifying such families. However, in the MelaNostrum example, it is the disease patterns that do not appear to have utility. One partial explanation is that, in this study, where each family had only an average of three cases, the disease patterns might not have varied enough as to be able to differentiate carriers of HPRV. Otherwise, we are left with conjectures, such that families may have misreported disease incidence and there are many HPRV beyond those in predefined genes. If a low mean PRS, as opposed to a high T, continues to be a better predictor of the presence of an HPRV in future studies, the simpler statistic might be a better way to select families. Note, the superiority of the PRS statistic might be possible, if not likely, if identifying diseased individuals in extended families is prone to error.

We showed some extreme examples where simple case-control studies had higher statistical power when only using the subset of cases with the highest values of T_i_, as opposed to all cases. In such scenarios, we could clearly gain power by using all cases if we modified the statistical test. For example, we might compare the proportion of controls carrying an HPRV to a weighted average of the genotypes in the affected family members, where the weights are lower for individuals with higher PRS. As sequencing costs continue to decrease and studies no longer need to select a limited number of individuals, weighting will become a more valuable use of PRS. Therefore, future investigations should evaluate the potential gains of weighting.

We are not the first to suggest using PRS to select individuals for sequencing studies and specifically built our approach upon the elegant ideas proposed by Jostins [17]. We extend prior ideas by introducing a new statistic that attempts to estimate the number of HPRVs, accounting for ungenotyped individuals by modeling the PRS as a multivariate normal distribution instead of imputing individual genotypes, and by considering the influence of the total polygenic heritability. We expect the performance of both statistics, as well as the simpler average PRS in a family, to be evaluated with actual data as it becomes available.

We have pointed out two main limitations of our study. First, both our predictions about the presence of an HPRV and the form of our test statistic rely on the liability threshold model. Although this model has been a staple of genetics for over 80 years [19], the model has never been evaluated for its accuracy in estimating the predictive capacity of polygenic risk scores. Second, without knowledge of either the number or penetrance of these rare causal variants, we cannot offer precise predictions about the true proportion of families carrying an HPRV or the utility of PRS in selecting families for sequencing. Finally, it is worth highlighting a general limitation of PRS. For most diseases, PRS were developed in European populations and their predictive accuracy will be lower in other populations. Therefore, the benefits of our proposed method for selecting afflicted families may be greatly diminished when the PRS was, in fact, developed in a European population but the study’s families have non-European ancestry. The performance of the method would be further comprised if the imputation quality of the imputed SNPs used in the PRS was poor because the families’ ancestries were not properly included on the imputation panel. Despite these limitations, we have proposed a new test statistic for identifying families with an HPRV and believe that the performance of the statistic may be worth exploring in future studies.

## Methods

We divide our Methods into four sections, describing the notation, the PRS-statistic, the scenarios for inquiry, and the WES study of Melanoma.

### Notation

We assume that there is a single highly penetrant rare variant (HPRV), but note that our framework holds for multiple HPRVs with appropriate modifications to the definitions (e.g. “*p*_*G*_” would be the combined frequency of all HPRVs).

*n*_*F*_ is the total number of families available for sequencing (*i* ∈ {1,…*n*_*F*_} indexes families).
*N*_*i*_ is the number of individuals in family *i* (*k* ∈ {1,…*N*_*i*_} will index family members).
*Ω*_*i*_ ⊂ {1, … *N*_*i*_} is the subset of diseased individuals to be sequenced.
*D*_*ik*_ ∈ {0,1} indicates the disease status; $D_{i}=\left\{D_{i1},\ldots ,D_{iN_{i}}\right\}$.
*G*_*ik*_ ∈ {0,1,2} indicates the genotype at the rare variant; $G_{i}=\left\{G_{i1},\ldots ,G_{iN_{i}}\right\}$.
*S*_*ik*_ is the Polygenic Risk Score (PRS); $S_{i}.=\left\{S_{i1},\ldots ,S_{iN_{i}}\right\}$.
*p*_*G*_ is the Minor Allele Frequency (MAF) of the variant defining *G*_*ik*_.
$\sigma_{S}^{2}=\text{var}(S_{ik})$ is the polygenic risk score heritability.
*π*. = {*π*_0_,*π*_1_,*π*_2_} are the prevalences/penetrances of disease in individuals with *G*_*ik*_ = 0, 1, and 2.
$\pi^{*}=\pi_{0}{\left(1-p_{G}\right)}^{2}+\pi_{1}2p_{G}\left(1-p_{G}\right)+\pi_{2}p_{G}^{2}$ is the overall prevalence of disease in the population.
$\sigma_{P}^{2}$ is the total polygenic heritability.

We are assuming that the liability threshold model, described in the next paragraph, is true and that the underlying disease liability (*L*_*ik*_) can be decomposed into a polygenic effect from identified sources (*S*_*ik*_), a polygenic effect from unidentified sources $\left(S_{ik}^{U}\right)$, and a non-genetic effect (*ϵ*_*ik*_) with $L_{ik}=S_{ik}+S_{ik}^{U}+\epsilon_{ik}$ and the polygenic heritability defined by $\sigma_{P}^{2}=\mathrm{var}(S_{ik}+S_{ik}^{U})$.

### Liability threshold model

The disease liability and its three components follow multivariate normal distributions. Letting *I* be the *N*_*i*_ × *N*_*i*_ identity matrix and Σ_*i*_ be the kinship matrix for family i,

$$L_{i\cdot }=S_{i\cdot }+S_{i\cdot }^{U}+\epsilon_{i\cdot }$$

$$S_{i\cdot }\sim N\left(0,\sigma_{S}^{2}\Sigma_{i}\right)$$

$$S_{i\cdot }^{U}\sim N\left(0,\sigma_{U}^{2}\Sigma_{i}\right)$$

$$\epsilon_{i\cdot }\sim N\left(0,\sigma_{\epsilon }^{2}I\right)$$

where *S*_*i*_, $S_{i\cdot \cdot }^{U}\cdot$, and *ϵ*_*i*_ are independent of each other and $\sigma_{S}^{2}+\sigma_{U}^{2}+\sigma_{\epsilon }^{2}=1$.

The disease thresholds (*c*_*i*_.) depend on Genotypes (*G*_*i*_.) and population-level penetrances (*π*_0_, *π*_1_,*π*_2_). In other words, the disease threshold for a given person depends on whether that individual has a rare variant (i.e. in the presence of a rare variant, liability need not be as large for the disease to occur). Letting Φ be the cumulative distribution for the standard normal distribution, define $c_{g}=\Phi^{-1}(1-\pi_{g})\;\text{and}\;c_{i}{}_{k}=c_{G_{i}{}_{k}}$. Then, the liability threshold model states that the disease status for an individual is determined by

$$D_{ik}=\{\begin{array}{c} 1\;if\;L_{ik}\geq c_{ik}\\ 0\;if\;L_{ik}<c_{ik} \end{array}$$

### PRS statistic

We will define our PRS statistic so that families with a higher value are, by some measure, more likely to carry a risk allele at the rare variant under investigation.

For defining the statistic, let $X_{i}\equiv X_{i}\left(D_{i\cdot },G_{i\cdot }\right)\;=\sum_{k\in \Omega_{i}}G_{ik}$ be the total number of rare alleles among the *n*_*i*_ sequenced cases in a family and *V*_*i*_ = *var*_0_(*X*_*i*_) be the variance of *Xi* under the null hypothesis, when the variant does not affect disease. Then, we define the PRS-statistic for the i^th^ family to be

$$T_{i}=\frac{E[X_{i}|D_{i\cdot },S_{i\cdot }]}{\sqrt{n_{i}V_{i}}}$$

and show how to calculate *T*_*i*_ in the Supporting Information: S1 Doc, with the assumptions that the liability threshold model is true and all parameters are known. We show how to calculate *T*_*i*_ when only a subset of individuals have PRS information in the Supporting Information: S1 Doc. For comparison, we also consider the test statistic, $T_{i}^{*}$, that does not consider PRS, $T_{i}^{*}=E[X_{i}|D_{i\cdot }]/\sqrt{n_{i}V_{i}}$.

We offer some intuition behind our test statistic *T*_*i*_, by rewriting the statistic as

$$T_{i}\propto \frac{E\left[X_{i}\text{|}D_{i\cdot },S_{i\cdot }\right]}{n_{i}}\times \frac{\sqrt{n_{i}p_{G}\left(1-p_{G}\right)}}{\sqrt{V_{i}}}$$

The first term on the right side is proportional to the expected number of HPRV per individual. The second term is the adjustment that accounts for the correlation of the genotypes among family members. In other words, we are not only interested in the average number of HPRV per individual, but we are interested in subsequent test statistic that accounts for the variance of that average.

### Scenarios

We describe the metrics and scenarios that we use to evaluate the characteristics of familial aggregation and study power. We estimate the metrics by simulating populations (and studies) according to the liability threshold model. We start by identifying the pedigree of interest, which is either the sibship or multi-generational family. Based on the genetic parameters (i.e. MAF, $\sigma_{S}^{2},\sigma_{p}^{2},p_{G}$), we then simulate > 50,000,000 pedigrees (i.e. *G*_*i⋅*_ *D*_*i⋅*_ *L*_*i⋅*_, *S*_*i⋅*_). Finally, we identify at least 10,000 families with the specified disease pattern and calculate their test statistics. From this group of families, we calculate all desired metrics.

We start by defining the three key metrics: *E*_*p*_, *β*(*q*_*T*_) and *M*(*q*_*T*_). Our first objective is to estimate the proportion, *E*_*p*_, of individuals in afflicted families that have at least one rare-variant

$$E_{p}=\text{E}[1(G_{ik}>0)|D_{ik}=1]$$

and show that *E*_*p*_ is not always close to 1. Our second objective is to estimate the power, *β*(*q*_*T*_), of a hypothetical study that selects the *q*_*T*_ × *n*_*F*_ families with the highest values of *T*_*i*_. Note, *n*_*F*_ is the total number of available families and, for our exposition, will be defined so that the study power is 0.6 when *q*_*T*_ = 1. This hypothetical study then sequences all diseased individuals from the selected families, calculates an estimate, ${\hat{p}}_{G}\left(q_{T}\right)$, of the MAF in those individuals, and tests for an association using the statistic $Z\left(q_{T}\right)\;=\left({\hat{p}}_{G}^{*}\left(q_{T}\right)\;-p_{G}^{*}\right)/\sqrt{var\left({\hat{p}}_{G}^{*}\left(q_{T}\right)\right)}$, where we assume the overall MAF accounts for 1000 rare variants, $p_{G}^{*}=p_{G}/1000$ and ${\hat{p}}_{G}^{*}={\hat{p}}_{G}/1000$. Then the power is defined by

$$\beta \left(q_{T}\right)\;=\text{Pr}(Z\left(q_{T}\right)\;>5.32)$$

where the threshold is chosen to correspond to a p-value of 5 ×10^-8^. As part of this second objective, we will also discuss the enrichment *M*(*q*_*T*_) of rare-variants in families at the top $q_{T}^{th}$ percentile of *T*_*i*_ among afflicted families

$$M\left(q_{T}\right)=\frac{\text{E}[1(G_{ik}>0)|D_{ik}=1,T_{i}=F_{T}^{-1}(1-q_{T})]}{\text{E}[1(G_{ik}>0)|D_{ik}=1]}$$

where *F*_*T*_ is the distribution function of *T*_*i*_.

We next define the scenarios for evaluating the three metrics. We consider two simple family structures (Fig 1). The two-generation family includes one founding couple with $n_{1}^{D}$ of their $n_{1}^{T}$ children having disease. The three-generation family has an additional $n_{2}^{D}$ of the $n_{2}^{T}$ grandchildren having disease, with the grandchildren evenly split among the children. Note, we only consider the individuals in the second and third generations (i.e. assume we cannot sequence the founding generation). We simulate either a large population of two-generation families with $n_{1}^{D}=n_{1}^{T}\in \left\{2,4,6\right\}$ or three generation families with $n_{1}^{T}=3,n_{2}^{T}=9$, and $n_{1}^{D}+n_{2}^{D}\in \left\{3,6,9\right\}$. We simulate these families assuming $\sigma_{P}^{2}\in \left\{0.15,0.30,0.45\right\}$, $\sigma_{S}^{2}\in \left\{0.1,0.3\right\}$, $p_{g}\in \left\{0.00001,0.0001,0.001,0.01\right\}$, and $\pi^{*}\in \left\{0.005,0.02,0.05\right\}$. We acknowledge that an allele with MAF = 0.01 would not be considered rare, but this MAF could represent the combined frequency of 1000’s of rare variants. For all scenarios, *π*_1_ = 0.5, and *π*_2_ = 0.5.

### MelaNostrum consortium

We considered melanoma-prone families participating in the MelaNostrum consortium. As part of this consortium, 6961 affected individuals from Italy, Spain, and Greece and 5553 controls were successfully genotyped using the Illumina OmniExpress Array. Moreover, a select subcohort of cases from 404 melanoma-prone families successfully received Whole Exome Sequencing (WES) or had been previously identified as carrying a *CDKN2A* mutation. Full details of genotyping, sequencing, and family selection have been described elsewhere [20, 21] and a brief summary is also provided in the Supporting Information: S1 Doc. For the PRS, we used the SNPs previously identified in the MelaNostrum consortium [22]. Of the 204 identified SNPs, 193 were carried by the subgroup of cases from the melanoma-prone families. We multiplied the genotype, coded as 0/1/2, by the beta coefficient for these SNPs and calculated the PRS by subtracting off the mean of the country-specific controls and dividing by the standard deviation in those same controls. For reference, we approximated the PRS heritability of melanoma in the Italy and Spain cohorts to be respectively 0.03 and 0.05 by performing linear regression with disease status and PRS as the dependent and independent variables, obtaining the coefficient ${\hat{\beta }}_{PRS}$ and estimating $\sigma_{S}^{2}\;\text{by}\;1-var(R_{i})/var(D_{i})$ where $R_{i}=D_{i}-{\hat{\beta }}_{PRS}S_{i}$. We then identified the potential Highly Penetrant Rare Variants (HPRV) in one of 7 genes (*CDKN2A*, *CDK4*, *BAP1*, *TERT*, *POT1*, *ACD*, *TERF2IP*) previously associated with melanoma [23]. After creating a list of all variants passing QC filters, we removed variants meeting any of the following criteria: 1) Minor Allele Frequency (MAF) > 0.001 in ExAC Non-Finish Europeans, MAF > 0.001 in both ExAC and the 1000 genome database, or MAF > 0.001 in our in-house Eagle controls (same population). 2) Listed as low/modifier consequences mutations ("3'UTR", "5'UTR", "3'Flank", "Targeted_Region", "Silent", "Intron", "RNA", "IGR", "Splice_Region", "5'Flank", "lincRNA"). 3) In WES samples, a note of “CScoreFilter” under FILTER column in VCF files or a genotype called as “0/0”. We note that 33 variants were missense mutations, while the remaining three included two frameshift deletions (*CDKN2A*, *ACD*) and one nonsense mutation (*POT1*).

## Supporting information

S1 Doc This word document contains an expanded methods sections detailing genotyping and sequencing procedures, a discussion about the properties of the liability-threshold model, and results from additional simulations. (DOCX)

S1 Tab This .txt file lists the SNPs and their coefficients used for calculating the polygenic risk score. (TXT)

S2 Tab This .txt files lists the rare potentially deleterious variants identified in the MelaNostrum families. (TXT)

S1 Fig The relative risk of disease as a function of the PRS score. Note that the liability threshold model has interesting implications for polygenic risk scores (PRS). In brief, individuals in the population with a low PRS have an extremely low risk of disease. If the disease has a prevalence of 0.01, then the relative risk of disease (e.g. probability of disease divided by 0.01) in individuals at the bottom 10^th^ percentile are approximately 0.01, 0.2, and 0.4 for PRS heritabilities of 0.05, 0.1, and 0.3. Moreover, the total heritability is irrelevant to these calculations. These results emphasize that even when the polygenic risk scores account for minimal heritability, individuals in the lowest percentiles are at a greatly reduced risk of disease.
(TIF)

S2 FigProportion carrying HPRV in affected sibships.The proportion (E_p_) of individuals carrying a highly penetrant rare variant (HPRV) in affected sibships for a disease with a 0.5%, 2% or 5% prevalence (100 × π*) in the population. Columns represent different values of polygenic heritability and rows represent different prevalences. Within a panel, the four sets of bars represent different MAF (p_G_ = 0.00001, 0.0001, 0.001, or 0.01; note, the number in parenthesis is the resulting Sibling Relative Risk). Within a set of bars, the colors represent different the number of children (red: $n_{1}^{T}\;=\;n_{1}^{D}\;=\;$2, purple: $n_{1}^{T}\;=\;n_{1}^{D}\;=\;$4, blue: $n_{1}^{T}\;=\;n_{1}^{D}\;=\;6$).(TIF)

S3 FigProportion carrying HPRV in multi-generational families.The proportion (E_p_) of individuals carrying a highly penetrant rare variant (HPRV) in affected multi-generational families for a disease with a 0.5%, 2%, or 5% prevalence (100 × π*) in the population. Columns represent different values of polygenic heritability and rows represent different prevalences. Within a panel, the four sets of bars represent different MAF (p_G_ = 0.00001, 0.0001, 0.001, or 0.01; note, the number in parenthesis is the resulting Sibling Relative Risk). Within a set of bars, the colors represent different the numbers of total affected individuals (red: $n_{1}^{D}+n_{2}^{D}\;=\;3$, purple: $n_{1}^{D}+n_{2}^{D}\;=\;6$, blue: $n_{1}^{D}+n_{2}^{D}\;=\;9$).(TIF)

S4 FigEnrichment and power to detect HPRV by the PRS statistic.The first two columns show the enrichment (M) of HPRV as a function of the PRS statistic when a sibship has four affected individuals and when a multi-generational family has six affected individuals. The X-axis is the quantile of the statistic (i.e. 0.1 represents a family at the top 10^th^ percentile). The last two columns show the power (β) to detect an association with the HPRV when a sibship has four affected individuals and when a multi-generational family has six affected individuals. The X-axis the quantile of the statistic (i.e. 0.1. represents a study where we select the 10% of affected families with the highest PRS statistic). In all four panels, the color indicates the PRS heritability (red, purple, blue indicates $\sigma_{S}^{2}$ = 0.3, 0.1, and 0.05 respectively) and the polygenic heritability is fixed at $\sigma_{P}^{2}$ = 0.3. The MAF and disease prevalence are listed at the top of each graph.(TIF)
