# Supplementary material for: Contribution of Common Genetic Variants to Familial Aggregation of Disease and Implications for Sequencing Studies
Source: PLoS Genet. 2019 Nov 15;15(11):e1008490. doi: 10.1371/journal.pgen.1008490 (PMC6881075; doi:10.1371/journal.pgen.1008490)
Supplement: S1 Doc — (DOCX) [file pgen.1008490.s001.docx]

**Supplementary Methods:**

Genome-wide SNP genotyping and Whole-exome sequencing was performed at the Cancer Genomics Research Laboratory (CGR), Division of Cancer Epidemiology and Genetics (DCEG), National Cancer Institute (NCI).

*Genome-wide SNP genotyping*

Genotyping was performed using Infinium Global Screening arrays. Genotypes were called using default parameters in GenomeStudio (Illumina). Prior to imputation any SNP with either minor allele frequency (MAF) < 0.01, Hardy-Weinberg Equilibrium (HWE) P-value 5 < 10-4 in controls or 5 < 10-10 in cases was removed. Similarly, any individual was removed who was missing > 0.03 of variants, had heterozygosity values either > 0.05 or < -0.05 or 3 sd from the mean, whose genetically-predicted sex did not match their recorded sex, or who was determined to be non-European based on principal component analysis (PCA) was removed. Imputation was conducted using the Michigan Imputation Server with the Haplotype Reference Consortium panel (HRC version 1) and run using Minimac3. Following imputation, any imputed variant with imputation quality score r2 < 0.5 or MAF < 0.0001 was rejected. Logistic regression was then conducted using PLINK (v1.90b5.4) with principal components as covariates to account for potential population stratification. The coefficients from this logistic regression were then used to calculate the PRS.

*Whole-exome sequencing*

For each sample, 1.1 μg of genomic DNA extracted from blood was used for exome sequence capture, which was performed with SeqCap EZ Human Exome Library v2.0 or v3.0 (Roche NimbleGen). Pools of captured DNA then underwent paired-end sequencing using an Illumina HiSeq according to Illumina-provided protocols for 2 × 100-bp paired-end sequencing. Each exome was sequenced to high depth to achieve a minimum threshold of 80% of coding sequence covered with by least 15 reads, on the basis of the UCSC hg19 'known gene' transcripts. Sequencing reads were aligned to the hg19 reference genome using Novoalign software version 2.07.14. Variant discovery and genotype calling of multi-allelic substitutions, insertions and deletions was performed on all individuals globally using the UnifiedGenotyper module from the Genome Analysis Toolkit (GATK) with the minimum call quality parameter set to 30.

*Calculating the PRS-statistic*

Let us focus on the numerator and make the reasonable assumption that $G_{i\cdot}$ is independent of $S_{i\cdot}$

$$E\left[ X_{i}(D_{i\cdot},G_{i\cdot}) | D_{i\cdot},S_{i\cdot} \right]=\sum_{g} X_{i}(D_{i\cdot},g) Pr\left[ G_{i}=g | D_{i\cdot},S_{i\cdot} \right]=\sum_{g} X_{i}(D_{i\cdot},g)\frac{Pr(g,D_{i\cdot}|S_{i\cdot})}{Pr(D_{i\cdot}|S_{i\cdot})}=$$

$$\sum_{g} X_{i}(D_{i\cdot},g)\frac{Pr(D_{i\cdot}|g,S_{i\cdot})Pr(g|S_{i\cdot})}{Pr(D_{i\cdot}|S_{i\cdot})}=\sum_{g} X_{i}(D_{i\cdot},g)\frac{Pr(D_{i\cdot}|g,S_{i\cdot})Pr(g)}{Pr(D_{i\cdot}|S_{i\cdot})}$$

Now, we calculate $\Pr\left( D_{i\cdot} | g,S_{i\cdot} \right)$ by assuming the liability-threshold model holds. Specifically, for a given genotype $g=(g_{1},\ldots,g_{N_{i}})$, we define the region $R_{i}\left( g \right)=R_{i}^{1}\left( g \right)\times R_{i}^{2}\left( g \right)\times\ldots\times R_{i}^{N_{i}}\left( g \right)\subset\mathfrak{R}^{N_{i}}$ where $R_{i}^{k}\left( g \right)=(-\infty,c_{g_{k}})$ if $D_{ik}=0$ and $R_{i}^{k}\left( g \right)=(c_{g_{k}},\infty)$ if $D_{ik}=1$. Furthermore, let $\phi(\mu,\Sigma)$ denote the density of a multivariate normal distribution with the specified mean and variance. Then

$$\Pr\left( D_{i\cdot} | g,S_{i\cdot} \right)=\int_{R_{i}\left( g \right)} \phi(S_{i\cdot},{(\sigma_{P}^{2}-\sigma_{S}^{2})\Sigma}_{i}+({1-\sigma}_{P}^{2})I_{N\times N})$$

For large families, integrating over all genotypes is intractable. In these families, we assume that one of the common founders (i.e. ancestor of all cases) has a single risk variant and all other founders have none.

Under Hardy-Weinberg-Equilibrium the denominator of the statistics is given by $V_{i}={2p}_{G}\left( 1-p_{G} \right)\sum_{l,m} \Sigma_{i}[l,m]$ where $\Sigma_{i}[l,m]$ is $(l,m)^{th}$ entry of the kinship matrix (i.e. $\sum_{l,m} \Sigma_{i}[l,m]$ is the sum of all elements in the kinship matrix).

*Calculating the PRS-statistic with missing PRS*

In “*Calculating the PRS-statistic”*, we assumed that all individuals had a PRS. When some individuals have missing PRS values, we estimate the numerator of $T_{i}$ as follows.

Let $A_{i\cdot}=\{A_{i1},\ldots,A_{iN}\}$ be binary variables indicating available PRS for N individuals in family i.

Let $S_{i1\cdot}=\{S_{ik}:A_{ik}=1\}$ ; $S_{i0\cdot}=\{S_{ik}:A_{ik}=0\}$; $S_{i\cdot}=\{S_{i1\cdot},S_{i0\cdot}\}$

Let us make the further reasonable assumption that $A_{i\cdot}$ is independent of genotype and PRS conditional on disease status. Then

$$E\left[ X_{i}\left( D_{i\cdot},G_{i\cdot} \right) | D_{i\cdot},S_{i1\cdot},A_{i} \right]=\sum_{g} X_{i}(D_{i\cdot},g) Pr\left[ G_{i}=g | D_{i\cdot},A_{i\cdot},S_{i1\cdot} \right]=\sum_{g} X_{i}\left( D_{i\cdot},g \right)\frac{\Pr\left[ D_{i\cdot},A_{i\cdot} | G_{i}=g,S_{i1\cdot} \right]\Pr\left[ g | S_{i1\cdot} \right]}{\Pr\left[ D_{i\cdot},A_{i\cdot} | S_{i1\cdot} \right]}=\sum_{g} X_{i}\left( D_{i\cdot},g \right)\frac{\Pr\left[ A_{i\cdot} | D_{i\cdot} \right]\Pr\left[ D_{i\cdot} | G_{i}=g,S_{i1\cdot} \right]\Pr\left[ g | S_{i1\cdot} \right]}{\Pr\left[ A_{i\cdot} | D_{i\cdot} \right]\Pr\left[ D_{i\cdot} | S_{i1\cdot} \right]}=\sum_{g} X_{i}(D_{i\cdot},g)\frac{Pr[D_{i\cdot}|G_{i}=g,S_{i1\cdot}]Pr[g|S_{i1\cdot}]}{Pr[D_{i\cdot}|S_{i1\cdot}]}=\sum_{g} X_{i}(D_{i\cdot},g)\frac{Pr[D_{i\cdot}|G_{i}=g,S_{i1\cdot}]Pr[g]}{Pr[D_{i\cdot}|S_{i1\cdot}]}$$

Next, assume that the subjects have been ordered so that individuals with a PRS precede those without a PRS. Furthermore, let $\Sigma_{i1}$ and $\Sigma_{i0}$ be the kinship matrices for individuals with $A_{ik}=1$ and $A_{ik}=0$ respectively and let by $\Sigma_{i10}$ be bottom-left submatrix of $\Sigma_{i}$. Let us then define $\Sigma_{i}^{*}$ to be the $N_{i}\times N_{i}$ matrix with $\Sigma_{i0}-\Sigma_{i10}\Sigma_{i0}^{-1}\Sigma_{i10}^{'}$ in the bottom-right corner and 0’s in all other entries. Then

$$\Pr\left( D_{i\cdot} | g,S_{i1\cdot} \right)=\int_{R_{i}\left( g \right)} \phi\left( {(S}_{i1\cdot},\Sigma_{i10}\Sigma_{i1}^{-1}S_{i1\cdot} \right),{(\sigma_{G}^{2}-\sigma_{S}^{2})\Sigma}_{i}+\sigma_{S}^{2}\Sigma_{i}^{*}+({1-\sigma}_{G}^{2})I_{N\times N})$$

The denominator is the same as in ‘*Calculating the PRS-statistic’*.
