# Supplementary figures and images for: Contribution of Common Genetic Variants to Familial Aggregation of Disease and Implications for Sequencing Studies
Source: PLoS Genet. 2019 Nov 15;15(11):e1008490. doi: 10.1371/journal.pgen.1008490 (PMC6881075; doi:10.1371/journal.pgen.1008490)

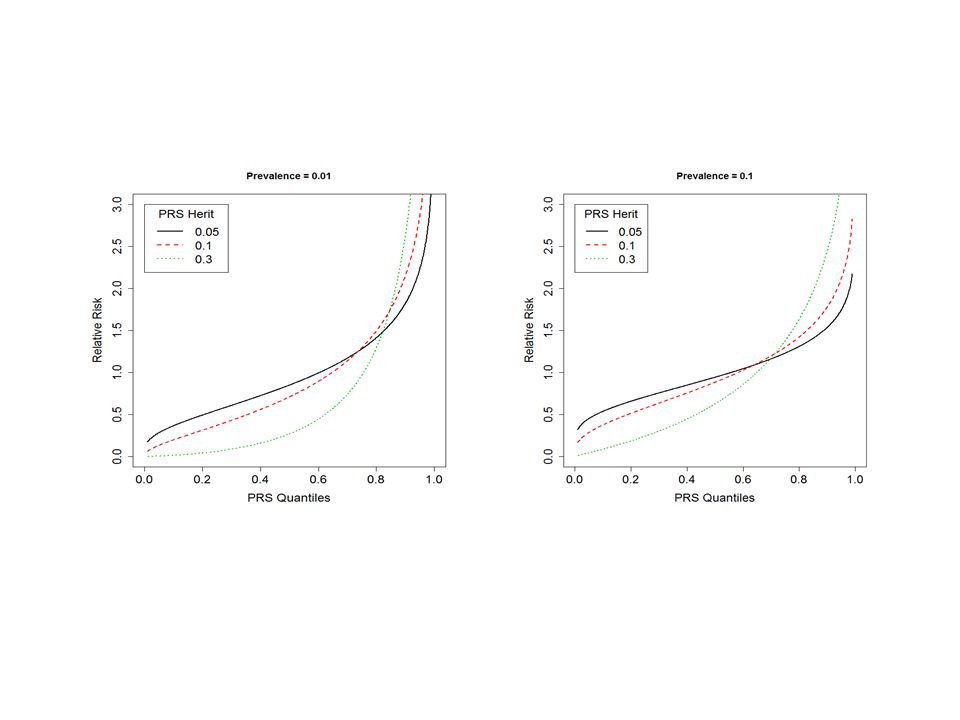

Supplement: S1 Fig — Note that the liability threshold model has interesting implications for polygenic risk scores (PRS). In brief, individuals in the population with a low PRS have an extremely low risk of disease. If the disease has a prevalence of 0.01, then the relative risk of disease (e.g. probability of disease divided by 0.01) in individuals at the bottom 10th percentile are approximately 0.01, 0.2, and 0.4 for PRS heritabilities of 0.05, 0.1, and 0.3. Moreover, the total heritability is irrelevant to these calculations. These results emphasize that even when the polygenic risk scores account for minimal heritability, individuals in the lowest percentiles are at a greatly reduced risk of disease. (TIF) [file pgen.1008490.s004.tif]

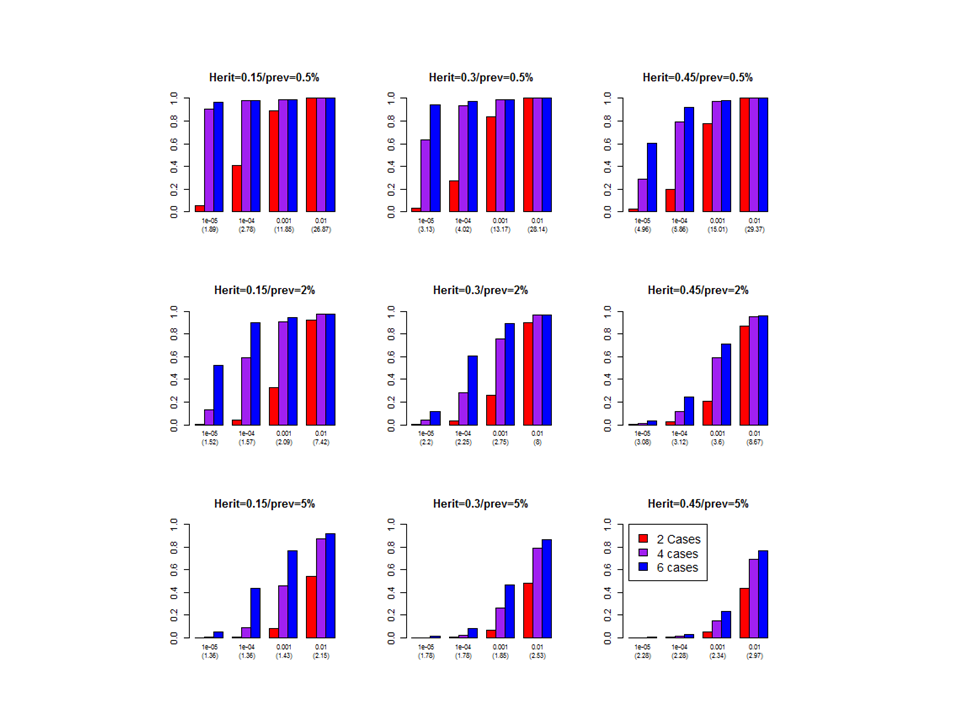

Supplement: S2 Fig — The proportion (Ep) of individuals carrying a highly penetrant rare variant (HPRV) in affected sibships for a disease with a 0.5%, 2% or 5% prevalence (100 × π*) in the population. Columns represent different values of polygenic heritability and rows represent different prevalences. Within a panel, the four sets of bars represent different MAF (pG = 0.00001, 0.0001, 0.001, or 0.01; note, the number in parenthesis is the resulting Sibling Relative Risk). Within a set of bars, the colors represent different the number of children (red: n1T = n1D = 2, purple: n1T = n1D = 4, blue: n1T = n1D = 6). (TIF) [file pgen.1008490.s005.tif]

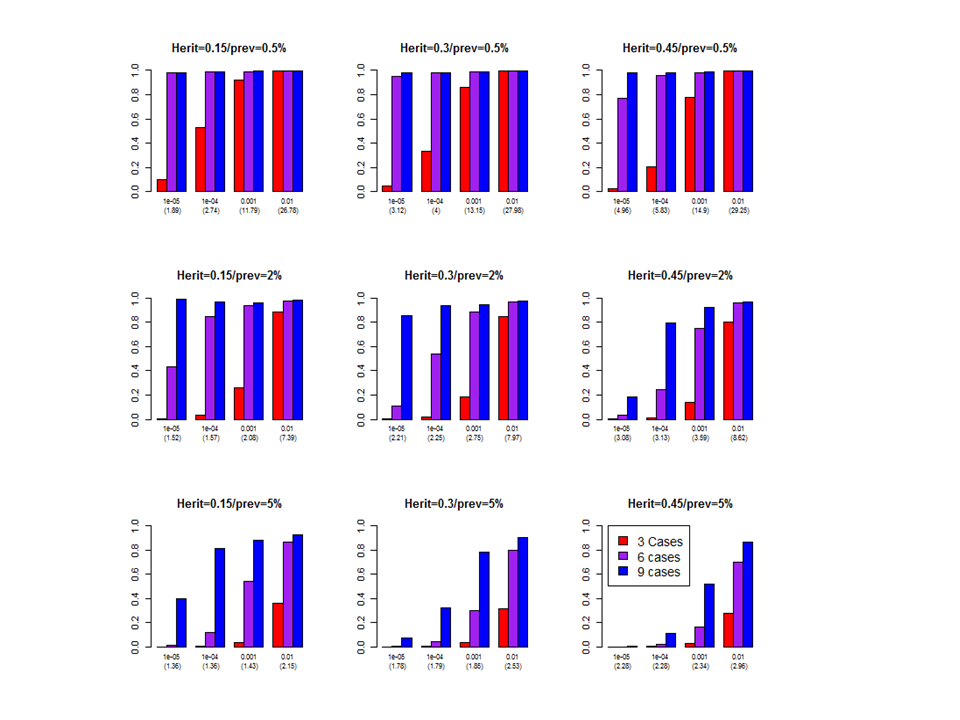

Supplement: S3 Fig — The proportion (Ep) of individuals carrying a highly penetrant rare variant (HPRV) in affected multi-generational families for a disease with a 0.5%, 2%, or 5% prevalence (100 × π*) in the population. Columns represent different values of polygenic heritability and rows represent different prevalences. Within a panel, the four sets of bars represent different MAF (pG = 0.00001, 0.0001, 0.001, or 0.01; note, the number in parenthesis is the resulting Sibling Relative Risk). Within a set of bars, the colors represent different the numbers of total affected individuals (red: n1D+n2D = 3, purple: n1D+n2D = 6, blue: n1D+n2D = 9). (TIF) [file pgen.1008490.s006.tif]

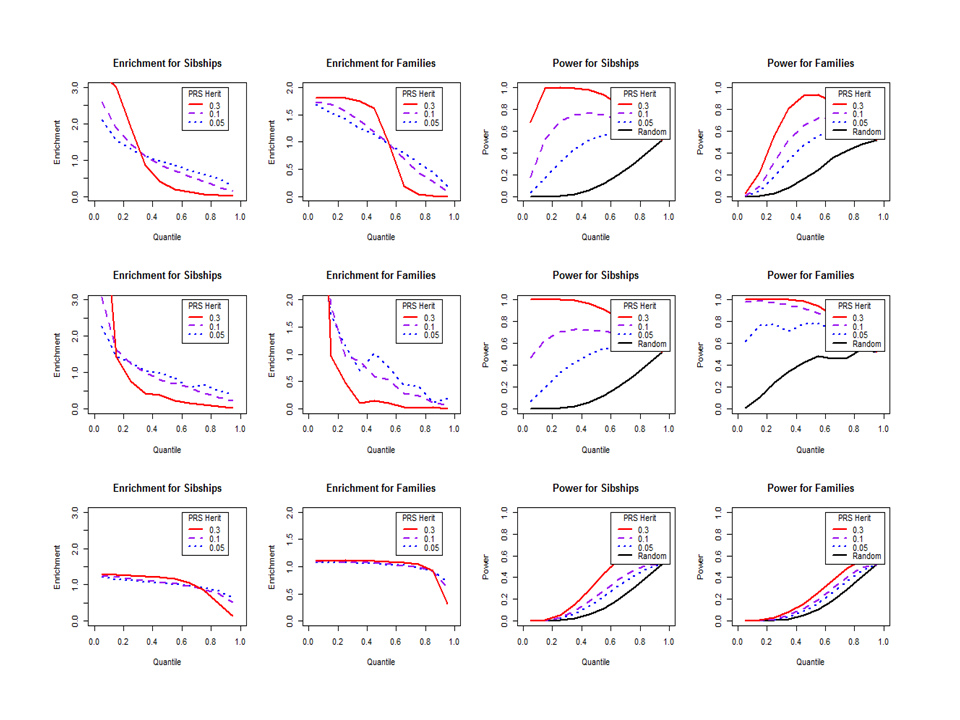

Supplement: S4 Fig — The first two columns show the enrichment (M) of HPRV as a function of the PRS statistic when a sibship has four affected individuals and when a multi-generational family has six affected individuals. The X-axis is the quantile of the statistic (i.e. 0.1 represents a family at the top 10th percentile). The last two columns show the power (β) to detect an association with the HPRV when a sibship has four affected individuals and when a multi-generational family has six affected individuals. The X-axis the quantile of the statistic (i.e. 0.1. represents a study where we select the 10% of affected families with the highest PRS statistic). In all four panels, the color indicates the PRS heritability (red, purple, blue indicates σS2 = 0.3, 0.1, and 0.05 respectively) and the polygenic heritability is fixed at σP2 = 0.3. The MAF and disease prevalence are listed at the top of each graph. (TIF) [file pgen.1008490.s007.tif]
